# What Changed on the Folliculogenesis in the Process of Mouse Ovarian Aging?

**DOI:** 10.1155/2019/3842312

**Published:** 2019-04-01

**Authors:** Wenlei Ye, Wei Shen, Wei Yan, Su Zhou, Jing Cheng, Guangxin Pan, Meng Wu, Lingwei Ma, Aiyue Luo, Shixuan Wang

**Affiliations:** ^1^Department of Obstetrics and Gynecology, Tongji Hospital, Tongji Medical College, Huazhong University of Science and Technology, Wuhan, Hubei, China; ^2^Department of Obstetrics and Gynecology, Zhongnan Hospital of Wuhan University, Wuhan University, Wuhan, Hubei, China; ^3^Department of Obstetrics and Gynecology, The Central Hospital of Wuhan, Tongji Medical College, Huazhong University of Science and Technology, Wuhan, Hubei, China

## Abstract

There are about 1-2 million follicles presented in the ovary at birth, while only around 1000 primordial follicles are left at menopause. The ovarian function also decreases in parallel with aging. Folliculogenesis is vital for ovarian function, no matter the synthesis of female hormones or ovulation, yet the mechanisms for its changing with increasing age are not fully understood. Early follicle growth up to the large preantral stage is independent of gonadotropins in rodents and relies on intraovarian factors. To further understand the age-related molecular changes in the process of folliculogenesis, we performed microarray gene expression profile analysis using total RNA extracted from young (9 weeks old) and old (32 weeks old) mouse ovarian secondary follicles. The results of our current microarray study revealed that there were 371 (≥2-fold, q-value ≤0.05) genes differentially expressed in which 174 genes were upregulated and 197 genes were downregulated in old mouse ovarian secondary follicles compared to young mouse ovarian secondary follicles. The gene ontology and KEGG pathway analysis of differentially expressed genes uncovered critical biological functions such as immune system process, aging, transcription, DNA replication, DNA repair, protein stabilization, and apoptotic process were affected in the process of aging. The considerable changes in gene expression profile may have an adverse influence on follicle quality and folliculogenesis. Our study provided information on the processes that may contribute to age-related decline in ovarian function.

## 1. Introduction

Ovarian aging results in the cessation of ovarian function, that is, anovulation and a decrease in gonadal steroids secretion. The anovulation causes loss of fertility and reduced hormone production results in multiple health consequences, including vasomotor symptoms, cardiovascular disease, osteoporosis, cognitive dysfunction, depression, mood disorders, sexual dysfunction, vaginal atrophy, and even mortality [1, 2]. The age at which natural menopause occurs may be a marker of ovarian aging which is considered to be the multiple pacemakers [3].

Ovarian follicle is the basic unit of ovarian physiological function. After puberty, the periodic development of the ovarian follicles enables the ovary to produce female hormones to maintain secondary sexual characteristics and ovulation. The reproductive aging process is considered to be dominated by the gradual decrease of both the quantity and the quality of the oocytes residing within the follicles present in the ovarian cortex [4]. Females have approximately 1-2 million primordial follicles at birth [5, 6]. After birth, the number of follicles decreases gradually with increasing age through atresia with some 300,000 to 400,000 primordial follicles remaining at menarche [4, 7]. During the reproductive years, the number of primordial follicles declines until a critical threshold when only about 1000 left at the time of menopause [8–10].

The information on the hormonal changes observed gradual decline of the follicle pool and the reduced oocyte quality during ovarian aging is quite a bit; however, the molecular mechanisms behind that are still not fully understood. Studies have shown that accumulation of reactive oxygen species (ROS) and free radicals and the action of environmental factors such as radiation and chemotherapeutic drugs used in cancer patients can cause DNA damage in the oocytes during long periods of dictyate arrest and without repairing, the extent of DNA damage may cause genomic abnormalities (chromosomal breakages and mutations) leading to cell death and follicle atresia [11, 12]. Researches have revealed that the expression of BRCA1 (breast cancer type 1) related DNA repair decreased in the process of ovarian aging in rat and buffalo primordial follicles [13, 14]. Laboratory and clinical studies also demonstrated that expression of BRCA1 declines in single mouse and human oocytes and BRCA1 mutation is associated with primary ovarian insufficiency [15–17].

A better understanding of follicle biology is essential to help make ovarian aging process explicit. Early follicle growth up to the large preantral stage is independent of gonadotropins in rodents and relies on intraovarian factors [6]. Thus in our present study, the secondary follicles from young and old mice ovaries were used to investigate the changes of expression profile during ovarian aging by genome-wide microarray analysis.

## 2. Materials and Methods

### 2.1. Isolation of Secondary Follicles from the Mouse Ovary

The experimental animals were maintained as per the guidelines of the Animal Care Committee of Tongji Hospital within the Tongji Medical College at the Huazhong University of Science and Technology in China. The 9-week old and 32-week old, specific pathogen-free (SPF), female C57BL/6J mice were obtained from Beijing Vital River Laboratory Animal Technology Co., Ltd. (Beijing, China). All mice were killed by decapitation and ovaries were collected free of adhering tissue. Under the stereomicroscope, follicles with diameter of 120-140 *μ*m, an intact basal membrane, a central and spherical oocyte surrounded by granulosa cells were mechanically dissecting by 2 syringe needles. The ovarian secondary follicles were stored at −80°C.

### 2.2. RNA Isolation and Microarray Analysis

The total RNA of the ovarian secondary follicles was extracted with RNAiso plus reagent according to the manufacturer's instructions (Takara, Japan). Of the total of 6 samples, 3 replicate samples were from young mouse ovarian secondary follicles and 3 replicate samples were from old mouse ovarian secondary follicles. All RNA samples were stored in DEPC in order to prevent RNA degeneration. GeneChip hybridization for each sample was examined on Affymetrix 3' IVT Expression Arrays (Mouse Genome 430 2.0 array) at Bioassay Laboratory of CapitalBio Corporation. The technical procedures and quality controls were performed at the CapitalBio Corporation. Hybridization assay procedures were as described in the GeneChip Expression Analysis Technical Manual (http://www.affymetrix.com).

### 2.3. Microarray Data Analysis

The raw data from microarray analysis was normalized using robust multiarray average (RMA) algorithm. The differentially expressed genes with a fold change ≥2 and a q-value ≤0.05 were identified using Significant Analysis of Microarray (SAM) software. For visualization of differentially expressed genes, unsupervised hierarchical clustering was performed using HemI 1.0.3.7 software (http://hemi.biocuckoo.org/down.php) [18]. Gene Ontology (GO) consisting of three items: molecular functions, biological processes, and cellular components [19] and Kyoto Encyclopedia of Genes and Genomes (KEGG), a set of high-throughput genes and protein pathways [20], analyses of differentially expressed genes were performed using the DAVID online tools (https://david.ncifcrf.gov/) compared with the mouse whole genome [21]. Whole Mouse Genome was used as the reference group. Statistical significance was calculated with a standard hypergeometric equation corrected by a Benjamini Yekutelli correction for multiple testing, which takes into account the dependency among the GO categories. The minimal length of considered GO-paths was 2. Significance was set at corrected p-value < 0.05. The Search Tool for the Tetrieval of Interacting Genes (STRING) database (http://string-db.org/), an online software that provides comprehensive interactions of lists of proteins and genes, was used to build a PPI network of the differentially expressed genes [22]. The cut-off criteria of the minimum required interaction score were 0.7 for the PPI network. The visualizing of the PPI network was constructed using the Cytoscape software (version 3.6.1) [23]. The Clustering with Overlapping Neighborhood Expansion (ClusterONE) plug-in for Cytoscape was used to detect protein complexes in the PPI network [24]. The gene regulatory network modeling for selected differentially expressed genes was performed using Cytoscape software (version 3.6.1).

## 3. Results

### 3.1. Global Gene Expression Analysis of Secondary Follicles from Mouse Ovaries

**Figure 1:**
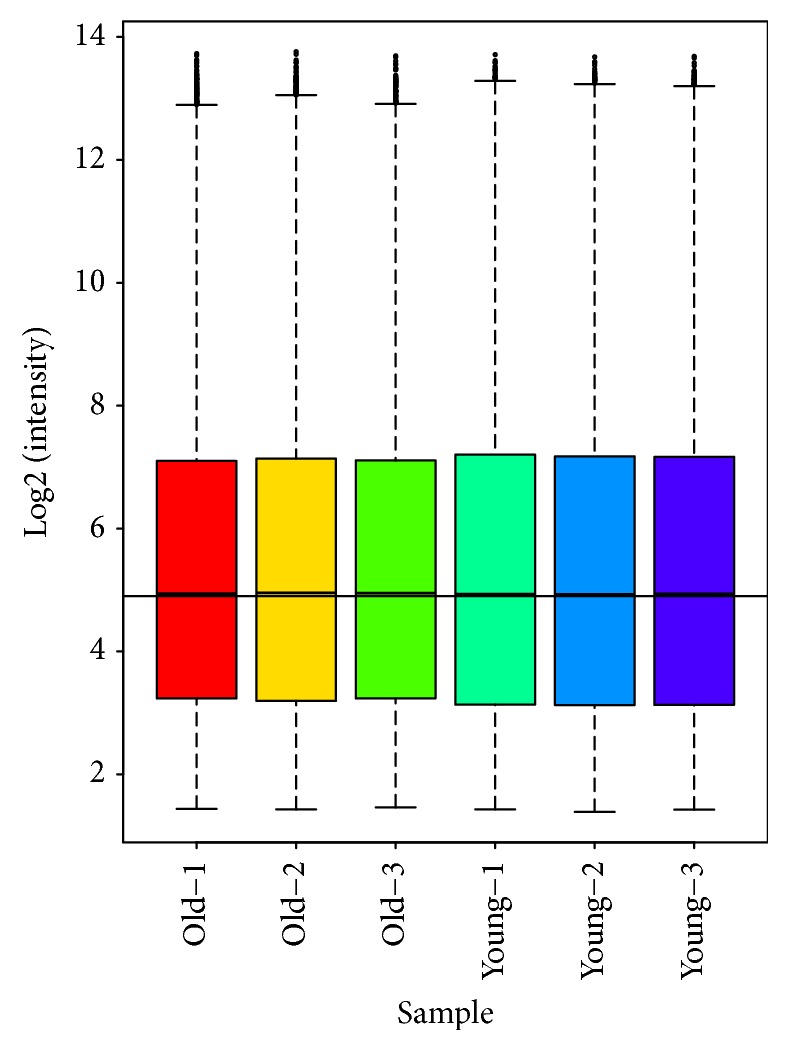
Box and whisker plot (Box plot). We performed the comparison of gene expression with a total of six samples from young (n=3) and old (n=3) mouse ovarian secondary follicles by using Affymetrix 3' IVT Expression Arrays (Mouse Genome 430 2.0 array) at Bioassay Laboratory of CapitalBio Corporation. Robust multiarray average (RMA) algorithm was used to eliminate the variation in the arrays from noisy data. Box plot was constructed to illustrate the distribution of normalized probe hybridization signal intensities (log ratios) for all six arrays (young and old mouse ovarian secondary follicles). The probe distribution by 0-100% quantiles as whiskers, the 25-75% quantiles as different color boxes, and the 50% quantile as horizontal line within the box indicated a similar range of signal intensities and confirmed perfect hybridization.

**Figure 2:**
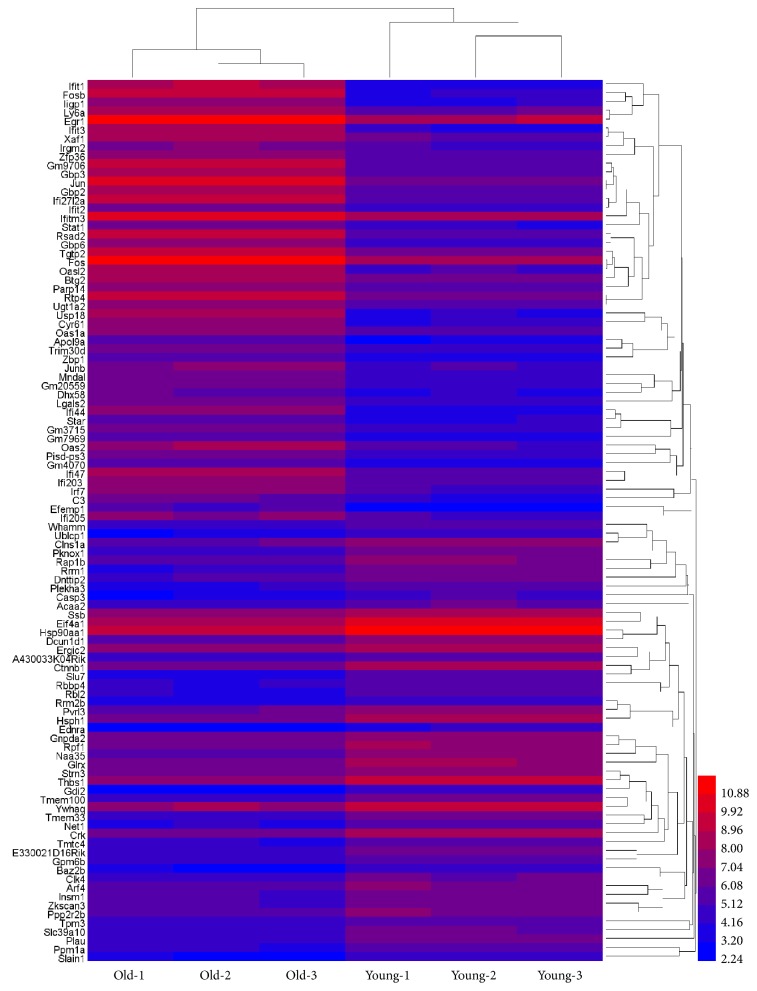
Heat map visualization for selected differentially expressed genes. Heat map was produced by unsupervised hierarchical-clustering analysis from microarray data for top 50 upregulated genes and top 50 downregulated genes in old mouse ovarian secondary follicles compared to young. The relative expression levels of each gene are mentioned in different colors. The red lines represent high expression, while blue lines represent low expression. The numbers in the right side, corresponding to the different colors, represent the relative expression levels of each gene.

To characterize the genes that are associated with mouse ovarian aging, we examined the gene expression profile of secondary follicles from young and old mouse ovaries. The expression values of all the six samples (three samples each from young and old mouse ovaries) were normalized using the robust multiarray average (RMA) method. The results of our microarray data were made available in the public domain NCBI-GEO repository (accession ID: GSE121493). The box-whisker plot analysis of normalized data showed uniform distribution of the expression levels in both intra- and intersample manner indicating reliable hybridization (see Figure 1). Summary statistics showed effectiveness of quantile normalization as 50th percentile values were close to 4.9. After normalization of raw data for all three biological replicates, the R package significance analysis of microarray (SAM) was used to identify genes that are differentially expressed in secondary follicles from young and old mouse ovaries (fold change ≥2 or ≤0.5 and q-value ≤0.05). And the results revealed that 371 genes were differentially expressed between the two groups, while 174 genes were upregulated and 197 genes were downregulated in the secondary follicles from the old mouse ovaries compared to those from the young mouse ovaries. Further, unsupervised hierarchical clustering analysis using the HemI 1.0.3.7 software showed distinct patterns of up- and downregulated genes in the secondary follicles from young and old mouse ovaries (see Figure 2).

### 3.2. Functional Annotation for the Differentially Expressed Genes

The identified differentially expressed genes in the secondary follicles from the old mouse ovaries compared to those from the young mouse ovaries were further analyzed via gene ontology (GO) and KEGG pathway analysis using the DAVID online tool. As shown in Table 1, GO term enrichment analysis showed that the upregulated genes were significantly enriched in immune system process in the biological processes category, cytoplasm, and nucleus in the cellular component category and RNA and DNA binding in the molecular function category. While listed in Table 2, the functional annotation for the downregulated genes revealed that the most significant categories of biological process were involved in transcription and its regulation, cellular component was involved in nucleus and cytoplasm, and molecular function was involved in protein, RNA, and DNA binding. Furthermore, KEGG pathway analysis showed that most of the upregulated genes took part in virus related and Toll-like receptor signaling pathways, whereas downregulated genes mainly participated in PI3K-Akt signaling pathway and Adherens junction in Table 3.

### 3.3. Protein-Protein Interaction and Gene Regulation Network Analysis

**Figure 3:**
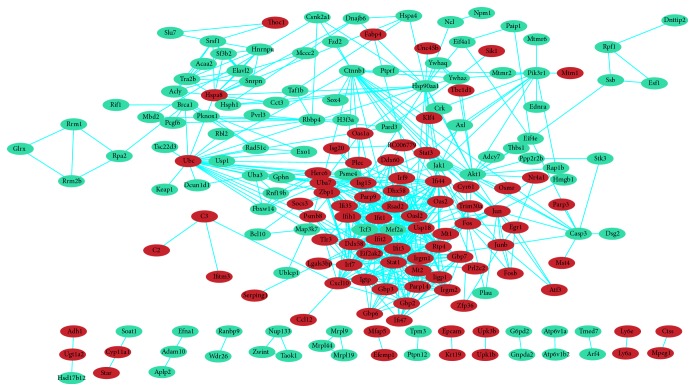
Protein-protein interaction (PPI) network complex. Using the Search Tool for the retrieval of Interacting Genes (STRING) database online database, 187 out of 371 differentially expressed genes (DEGs) (upregulated genes are displayed in red and downregulated genes in green) were filtered into the DEGs protein-protein interaction (PPI) network complex.

**Figure 4:**
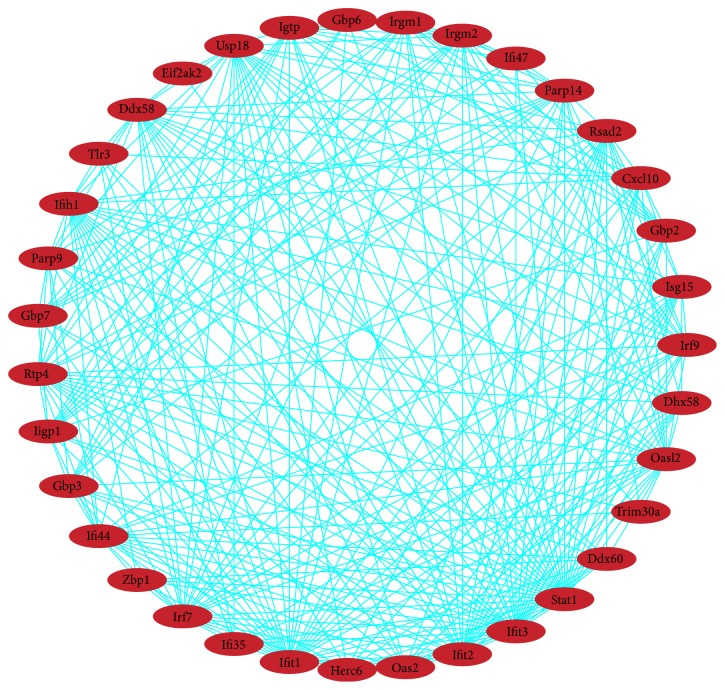
Overlapping protein complexes in the protein interaction. The most significant module consisted of 35 nodes and 286 edges, and all genes in this module were upregulated (in red).

**Figure 5:**
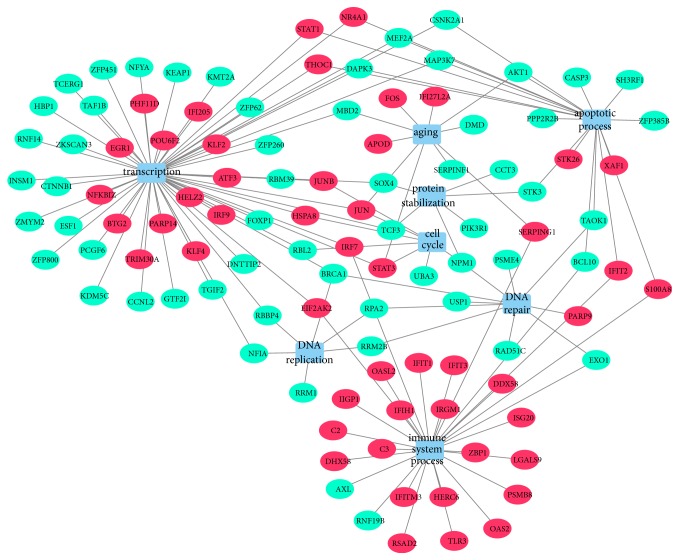
Gene regulatory network modeling for selected differentially expressed genes by using Cytoscape software (version 3.6.1). Gene regulatory network modeling of selected genes such as BRCA1, RPA2, STAT3, JUN, AKT1, STK3, SOX4, TCF3, S100A8, and IFIT2 showed their genetic interactions by various pathways. Circles indicate genes, red color indicates upregulation, and green color indicates downregulation.

In total, 187 nodes and 572 edges were mapped in the PPI network of identified differentially expressed genes using STRING with the minimum required interaction score > 0.7 (Figure 3). The 10 nodes with highest degree were regarded as hub genes: STAT1, IFIT1, IFIT3, IRF7, USP18, OASL2, IFIT2, UBC, DDX58, IFIH1. There were 12 modules generated by ClusterONE with p-value < 0.05. The most significant module with p-value < 0.001 contained 35 nodes and 286 edges (Figure 4). The 10 genes listed above apart from UBC were included in the module. In addition to the 9 genes, other nodes in the module were RSAD2, IFI47, TRIM30A, PARP14, PARP9, IFI44, RTP4, GBP7, ISG15, IRF9, GBP6, GBP3, IGTP, HERC6, IRGM1, DHX58, IIGP1, OAS2, DDX60, CXCL10, ZBP1, GBP2, EIF2AK2, IRGM2, IFI35, and TLR3. And all genes in the module were upregulated. As shown in the gene regulatory network modeling for selected genes, many differentially expressed genes such as BRCA1, STAT3, JUN, AKT1, SEPRING1, TCF3, MAP3K7, and IRF7 took part in various pathways (Figure 5).

## 4. Discussion

Elucidating the mechanism of ovarian aging has significant meanings to female health. The gradual decrease of both the quantity and the quality of the oocytes surrounded by the granulosa cells in all stages of follicles dominates the reproductive aging [4]. In previous study, Govindaraj et al. revealed that gene expression patterns changed considerably in the rat primordial follicles in the process of ovarian aging [25]. Folliculogenesis in the ovary is a highly dynamic and periodic process regulated by both intra- and extra-oocyte factors [26]. At each reproductive cycle, activated primordial follicles join the growing pool transiting to primary follicles [26]. Through further development, a primary follicle grows into a secondary follicle [26]. And these stages are gonadotropin independent, but depend on the complex bidirectional communication between the oocyte and the somatic cells [26]. In the subsequent stages of folliculogenesis, the presence of pituitary gonadotropins, follicle-stimulating hormone (FSH), and luteinizing hormone (LH) are required [26]. So the secondary follicles from the mouse ovaries were selected as research objective in our study.

Gene expression profile of the secondary follicles from the young and old mouse ovaries was compared by microarray analysis to find what changed in the process of ovarian aging in the present study. The results of our research found that 174 genes were upregulated and 197 genes were downregulated in the secondary follicles from the old mouse ovaries compared to those from the young mouse ovaries.

Further GO and KEGG pathway analyses were performed to study the function of the differentially expressed genes. The result of GO analysis showed that the upregulated genes were mainly involved in biological process such as immune system process and defense response, while downregulated genes were closely related to gene transcription and cell apoptosis. However, there was an unexpected phenomenon in the results of our functional enrichment. Many upregulated genes were involved in response to virus and interferon in the biological process and took part in several virus related signal pathway. This phenomenon revealed that the SPF mice used in our study might infect some viruses. However, the certain thing is that the immune related genes can be expressed in ovarian granulosa cells, not only in immune cells. Several earlier studies indicated that viruses can induce innate immune response in granulosa cells and perturb ovarian function in mouse [27, 28]. And immune response genes were overexpressed with increasing age as showed by several microarray studies of aging [29]. Recently the concept that innate immunity is an essential requisite in the ovulation process is forwarded [30]. The important role of innate immune cells in decreasing the senescence burden was also recognized [31]. There was a probability that innate immune related genes were upregulated in the process of aging and affected the progress of ovarian aging. Yet, the actual role of innate immunity in the process of ovarian aging or folliculogenesis needs to be further researched.

We conducted protein-protein interaction network analysis of differentially expressed genes. The nodes regarded as hub genes were mostly involved in innate immune system. As in the gene regulatory network, many differentially expressed genes between young and old mouse ovarian secondary follicles such as BRCA1, STAT3, JUN, AKT1, SEPRING1, TCF3, MAP3K7, and IRF7 showed their genetic interactions by various pathways. Thus, a number of pathways were interacted through many genes in the process of ovarian aging.

## 5. Conclusions

In conclusion, our data showed quite different gene expression patterns of the secondary follicles between young and old mouse ovaries. The differentially expressed genes involved in the process of ovarian aging are central to biological processes such as immune system process, aging, transcription, DNA replication, DNA repair, protein stabilization, and apoptotic process. However, many upregulated genes in the old mouse ovarian secondary follicles were innate immune related genes. We proposed that innate immune system may play a vital role in the process of ovarian aging. Our results of altered genes and related transcriptional networks may be helpful for understanding the mechanism of the folliculogenesis in the process of ovarian aging in mice.

There are however limitations in the present study. Findings of our present research were mainly based on the bioinformatics analysis and further experiments are needed to verify. Furthermore, these data were acquired with secondary follicles from mouse ovaries and needed to be confirmed with the samples from the human.

## Figures and Tables

**Table 1:** Functional enrichment analysis of upregulated genes in the old mouse ovarian secondary follicles compared to young mouse.

Category	Term	Count^*∗*^	%	P Value
GOTERM_BP_DIRECT	GO:0035458~cellular response to interferon-beta	15	8.6	5.62E-21
GOTERM_BP_DIRECT	GO:0009615~response to virus	18	10.3	6.73E-21
GOTERM_BP_DIRECT	GO:0051607~defense response to virus	21	12.1	1.15E-19
GOTERM_BP_DIRECT	GO:0002376~immune system process	24	13.8	1.01E-15
GOTERM_BP_DIRECT	GO:0045087~innate immune response	23	13.2	2.70E-14
GOTERM_BP_DIRECT	GO:0071346~cellular response to interferon-gamma	9	5.2	1.80E-08
GOTERM_BP_DIRECT	GO:0042832~defense response to protozoan	6	3.4	1.60E-06
GOTERM_BP_DIRECT	GO:0006952~defense response	8	4.6	1.36E-05
GOTERM_BP_DIRECT	GO:0032870~cellular response to hormone stimulus	6	3.4	1.57E-05
GOTERM_BP_DIRECT	GO:0034097~response to cytokine	7	4.0	1.79E-05
GOTERM_CC_DIRECT	GO:0020005~symbiont-containing vacuole membrane	5	2.9	1.46E-07
GOTERM_CC_DIRECT	GO:0005829~cytosol	28	16.1	7.00E-05
GOTERM_CC_DIRECT	GO:0005737~cytoplasm	65	37.4	6.61E-04
GOTERM_CC_DIRECT	GO:0005634~nucleus	58	33.3	2.75E-03
GOTERM_CC_DIRECT	GO:0048471~perinuclear region of cytoplasm	12	6.9	8.23E-03
GOTERM_CC_DIRECT	GO:0072562~blood microparticle	5	2.9	1.34E-02
GOTERM_CC_DIRECT	GO:0031225~anchored component of membrane	5	2.9	1.63E-02
GOTERM_CC_DIRECT	GO:0009897~external side of plasma membrane	7	4.0	2.23E-02
GOTERM_MF_DIRECT	GO:0003725~double-stranded RNA binding	9	5.2	2.62E-08
GOTERM_MF_DIRECT	GO:0003690~double-stranded DNA binding	11	6.3	3.33E-08
GOTERM_MF_DIRECT	GO:0003924~GTPase activity	11	6.3	1.81E-06
GOTERM_MF_DIRECT	GO:0005525~GTP binding	12	6.9	6.76E-05
GOTERM_MF_DIRECT	GO:0008134~transcription factor binding	10	5.7	5.97E-04
GOTERM_MF_DIRECT	GO:0003723~RNA binding	15	8.6	9.40E-04
GOTERM_MF_DIRECT	GO:0003677~DNA binding	25	14.4	1.58E-03
GOTERM_MF_DIRECT	GO:0001730~2′-5′-oligoadenylate synthetase activity	3	1.7	2.48E-03
GOTERM_MF_DIRECT	GO:0016817~hydrolase activity, acting on acid anhydrides	3	1.7	2.48E-03
GOTERM_MF_DIRECT	GO:0001077~transcriptional activator activity, RNA polymerase II core promoter proximal region sequence-specific binding	8	4.6	2.65E-03

^*∗*^Number of enriched genes in each term. The top ten terms based on P value were chosen in each category.

**Table 2:** Functional enrichment analysis of downregulated genes in the old mouse ovarian secondary follicles compared to young mouse.

Category	Term	Count^*∗*^	%	P value
GOTERM_BP_DIRECT	GO:0006355~regulation of transcription, DNA-templated	42	21.3	7.02E-05
GOTERM_BP_DIRECT	GO:0016311~dephosphorylation	8	4.1	9.84E-05
GOTERM_BP_DIRECT	GO:0006351~transcription, DNA-templated	35	17.8	3.19E-04
GOTERM_BP_DIRECT	GO:0045893~positive regulation of transcription, DNA-templated	16	8.1	5.56E-04
GOTERM_BP_DIRECT	GO:0030335~positive regulation of cell migration	9	4.6	8.87E-04
GOTERM_BP_DIRECT	GO:0006470~protein dephosphorylation	7	3.6	2.39E-03
GOTERM_BP_DIRECT	GO:0043154~negative regulation of cysteine-type endopeptidase activity involved in apoptotic process	5	2.5	4.21E-03
GOTERM_BP_DIRECT	GO:0030177~positive regulation of Wnt signaling pathway	4	2.0	5.61E-03
GOTERM_BP_DIRECT	GO:0043065~positive regulation of apoptotic process	10	5.1	5.85E-03
GOTERM_BP_DIRECT	GO:0097194~execution phase of apoptosis	3	1.5	6.92E-03
GOTERM_CC_DIRECT	GO:0005634~nucleus	106	53.8	3.23E-13
GOTERM_CC_DIRECT	GO:0005737~cytoplasm	100	50.8	4.49E-08
GOTERM_CC_DIRECT	GO:0005654~nucleoplasm	44	22.3	9.26E-08
GOTERM_CC_DIRECT	GO:0070062~extracellular exosome	50	25.4	3.19E-06
GOTERM_CC_DIRECT	GO:0043234~protein complex	19	9.6	3.26E-05
GOTERM_CC_DIRECT	GO:0071013~catalytic step 2 spliceosome	6	3.0	2.24E-03
GOTERM_CC_DIRECT	GO:0005925~focal adhesion	11	5.6	4.30E-03
GOTERM_CC_DIRECT	GO:0005829~cytosol	29	14.7	5.76E-03
GOTERM_CC_DIRECT	GO:0030529~intracellular ribonucleoprotein complex	9	4.6	1.17E-02
GOTERM_CC_DIRECT	GO:0005911~cell-cell junction	7	3.6	1.26E-02
GOTERM_MF_DIRECT	GO:0005515~protein binding	77	39.1	6.99E-09
GOTERM_MF_DIRECT	GO:0044822~poly(A) RNA binding	30	15.2	2.52E-06
GOTERM_MF_DIRECT	GO:0003723~RNA binding	20	10.2	3.71E-04
GOTERM_MF_DIRECT	GO:0003677~DNA binding	35	17.8	4.23E-04
GOTERM_MF_DIRECT	GO:0004725~protein tyrosine phosphatase activity	7	3.6	4.62E-04
GOTERM_MF_DIRECT	GO:0046982~protein heterodimerization activity	15	7.6	7.77E-04
GOTERM_MF_DIRECT	GO:0000166~nucleotide binding	35	17.8	9.78E-04
GOTERM_MF_DIRECT	GO:0016791~phosphatase activity	7	3.6	1.29E-03
GOTERM_MF_DIRECT	GO:0019903~protein phosphatase binding	6	3.0	2.02E-03
GOTERM_MF_DIRECT	GO:0005524~ATP binding	28	14.2	2.62E-03

^*∗*^Number of enriched genes in each term. The top ten terms based on P value were chosen in each category.

**Table 3:** Pathway enrichment analysis of differentially expressed genes in old mouse ovarian secondary follicles compared to young mouse.

Category	Term	Count^*∗*^	%	P value
Up-regulated				
KEGG_PATHWAY	mmu05164:Influenza A	15	8.6	2.25E-11
KEGG_PATHWAY	mmu05168:Herpes simplex infection	15	8.6	3.13E-10
KEGG_PATHWAY	mmu05162:Measles	11	6.3	6.23E-08
KEGG_PATHWAY	mmu05160:Hepatitis C	11	6.3	6.23E-08
KEGG_PATHWAY	mmu04380:Osteoclast differentiation	8	4.6	4.40E-05
KEGG_PATHWAY	mmu05161:Hepatitis B	8	4.6	1.12E-04
KEGG_PATHWAY	mmu04622:RIG-I-like receptor signaling pathway	6	3.4	1.58E-04
KEGG_PATHWAY	mmu04668:TNF signaling pathway	7	4.0	1.69E-04
KEGG_PATHWAY	mmu04620:Toll-like receptor signaling pathway	6	3.4	9.93E-04
KEGG_PATHWAY	mmu05133:Pertussis	5	2.9	2.38E-03
Down-regulated				
KEGG_PATHWAY	mmu04151:PI3K-Akt signaling pathway	14	7.1	4.14E-04
KEGG_PATHWAY	mmu05200:Pathways in cancer	14	7.1	1.31E-03
KEGG_PATHWAY	mmu04550:Signaling pathways regulating pluripotency of stem cells	8	4.1	1.69E-03
KEGG_PATHWAY	mmu04520:Adherens junction	6	3.0	2.03E-03
KEGG_PATHWAY	mmu04015:Rap1 signaling pathway	9	4.6	5.33E-03
KEGG_PATHWAY	mmu04390:Hippo signaling pathway	7	3.6	1.16E-02
KEGG_PATHWAY	mmu05145:Toxoplasmosis	6	3.0	1.36E-02
KEGG_PATHWAY	mmu04510:Focal adhesion	8	4.1	1.51E-02
KEGG_PATHWAY	mmu04022:cGMP-PKG signaling pathway	7	3.6	2.04E-02
KEGG_PATHWAY	mmu03040:Spliceosome	6	3.0	2.57E-02

^*∗*^Number of enriched genes in each term. The top ten terms based on P value were chosen in each category.

## Data Availability

The results of our microarray data were made available in the public domain NCBI-GEO repository.
